# Factors associated with the perceived barriers of health care access among reproductive-age women in Ethiopia: a secondary data analysis of 2016 Ethiopian demographic and health survey

**DOI:** 10.1186/s12913-020-05485-y

**Published:** 2020-07-25

**Authors:** Koku Sisay Tamirat, Zemenu Tadesse Tessema, Fentahun Bikale Kebede

**Affiliations:** 1 Department of Epidemiology and Biostatistics, Institute of Public Health, College of Medicine and Health Sciences, University of Gondar, Gondar, Ethiopia; 2 Amhara Regional State Health Bureau, Gondar, Ethiopia

**Keywords:** Perceived barriers, Healthcare access, Women, Ethiopia

## Abstract

**Background:**

Health care access is the timely use of personal health services to achieve the best health outcomes. Problems in accessing health care among reproductive-age may lead to various adverse health outcomes like death and disabilities. Therefore, this study aimed to identify factors associated with the perceived barriers of healthcare access among reproductive-age women in Ethiopia.

**Method:**

This study was based on secondary data sources from the 2016 Ethiopia Demography and Health Survey. The individual women record (IR) file was used to extract about 15, 683 women for the final analysis from the largest dataset. A composite variable of health care access was created from four questions used to rate health care access problems among women of reproductive age. To identify factors associated with the perceived barriers of health care access among reproductive-age women, generalized estimating equation (GEE) model was fitted. Crude and adjusted odds ratio (AOR) with a 95% confidence interval (CI) computed to assess the strength of association between independent and outcome variables.

**Results:**

This study revealed that the magnitude of perceived barriers of healthcare access among reproductive-age women was 69.9% with 95%CI (69.3 to 70.7) to at least one or more of the four reasons. Rural resident (AOR = 2.13, 95%CI: 1.79 to 2.53), age 35–49 years (AOR = 1.24, 95%CI: 1.09 to 1.40), divorced/separated (AOR = 1.34, 95%CI: 1.17 to 1.54), had no health insurance coverage (AOR = 1.19, 95%CI: 1.01 to 1.45), poorer (AOR = 2.09,95%CI: 1.86 to 2.35) and middle wealth (AOR = 1.57,95%CI:1.38 to 1.79), no education (AOR = 2.30, 95%CI:1.95 to 2.72), primary education (AOR = 1.84, 95%CI: 1.58 to 2.15) and secondary education (AOR = 1.31, 95%CI: 1.13 to 1.51) were factors associated with the perceived barriers of health care access.

**Conclusion:**

A significant proportion of women of reproductive age faced barriers to healthcare access, of which money and distance were the most frequently perceived barriers. Divorced/separated marital status, old age, rural dwelling, no health insurance coverage, low economic situation, and level of education were factors associated with perceived barriers. These findings suggest further strengthening and improving health care access to those women with low socio-economic status for the realization of universal health coverage.

## Background

**Fig. 1:**
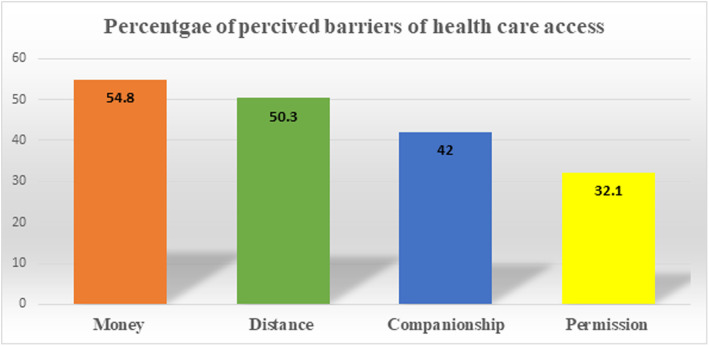
Percentage of perceived barriers of health care access among reproductive-age women in Ethiopia, 2016

In the past two decades, maternal health status showed a noteworthy improvement and achievements of Millennium Development Goals (MDGs), which was reflected by maternal and child mortality [1]. According to the 2015 MDGs report, maternal and under-five children mortality had decreased by 45 and 52%, respectively, from the 1990 baseline figs [1].. However, maternal health problems are still a significant concern and are unfinished agendas of MDG for low-income countries like Africa [1, 2]. Ethiopia is a highly affected country with the highest maternal and child mortality among sub-Saharan countries, with an estimated 353 deaths per 100,000 live births, according to the 2015 report [3]. Similarly, according to the Ethiopia 2016 national survey, institutional delivery was 66, and 22% of women had an unmet need for family planning services, which was linked to various barriers of accessibility and utilization [3]. Despite, all the United Nations (UN) member states have agreed to achieve universal health coverage by 2030 as part of SDGs; still half of the global population doesn’t have full coverage for essential health care services [4, 5].

Access to health care broadly defined based on availability, affordability, accessibility, and acceptability of services for best health outcomes [6]. Access to all-inclusive and quality health care is essential for promoting and maintaining health, preventing and managing the diseases, reducing unnecessary disabilities and premature deaths, and achieving health equity for all women [7–10]. Moreover, reproductive, maternal, newborn, and child health are indicators of the country’s socio-economic status and equitable o distribution of health care in the community [11–15].

It is known that literacy levels, economic conditions, socio-demographic and cultural characteristics, and geographical disparities play a vital role in affecting the accessibility and utilization of healthcare services among women [16–20]. Troubles conditions in accessing health care among reproductive-age women lead to diverse adverse health outcomes like unwanted pregnancies, unsafe abortion, maternal and child mortality resulting from low family planning uptakes, and home deliveries [7, 9, 14, 15, 21].

The Federal Democratic Republic of Ethiopia has a three-tier healthcare system, ranging from the lowest primary health care unit that provides essential health services to the highest tertiary hospitals for specialized services. Specifically, the primary health care units had components like health posts, which is mainly intended to provide essential health services (antenatal, postnatal and family dispenses, and immunization) to the population within five kilometers of radius by using health extension workers [22]. Establishing mobile maternal health clinics and expansion of health facilities and providing maternal health care services free of charge, are some of the interventions used to increase the service accessibility and utilization [23]. Literature showed that rural residents, low levels of education, financial hardship, and unemployment were factors associated with the challenges of health care access among reproductive-age women [12, 14–16, 18–20, 24]. A better understanding of the health care access difficulties using nationally representative data might assist in problem-solving and decision-making processes. Though many studies had been conducted to assess health care access challenges at different sites, no research has been to identify factors allied to difficulties of health care access among reproductive-aged women using national representative data.

Therefore, this study aimed to identify factors associated with the perceived barriers of healthcare access among reproductive-age women in Ethiopia. This study’s findings could help health care policymakers improve the health care of women through service redistribution to achieve equity of health care.

## Methods

### Data source

This study was based on secondary data analysis from the 2016 Ethiopia Demographic and Health Survey, which was collected cross-sectionally from January 18, 2016, to June 27, 2016. The study was conducted in Ethiopia, located in the Horn of Africa. The country has nine Regional states (Afar, Amhara, Benishangul-Gumuz, Gambela, Harari, Oromia, Somali, Southern Nations, Nationalities, and People’s Region (SNNP) and Tigray) and two Administrative Cities (Addis Ababa and Dire-Dawa).

### Population and samples

For the sampling purposes, the nine regional and two city administrations of Ethiopia were stratified into urban and rural enumeration areas, of which 202 and 443 EAs were initially selected from urban and rural areas, respectively, by probability sampling method. Then, a fixed number of 28 households per cluster were systematically selected. In the interviewed households, 16,583 eligible women identified for interviews of which, 15,683 women had completed the interview and included in the final analysis. The study participants from each EA stratum selected independently by using the probability sampling technique. The detail of the methodology is available in the full report of 2016 EDHS [3].

### Measurement of variables

Five data collection questionnaires were used for the 2016 EDHS, women’s questionnaire was one of the tools used to collect data about women and child health characteristics. For this study, women’s Individual Record (IR) file was used to extract, socio-demographic and reproductive traits were extracted from the most substantial dataset.

The Perceived barriers to health care access were the response variable. On the other hand, age, residence, wealth (economic) status, level of education, marital status, working status, health insurance coverage, and reproductive characteristics such as contraceptive use and intention, place of delivery, ANC follow up, and pregnancy during data collection were independent variables.

Each woman was interviewed to rate the difficulties of accessing health care based on obtaining money, health facility’s distance, permission to consult the doctor, and not wanting to go alone. Women reported at least one challenge of healthcare access (money, distance, companionship, and permission) considered as having perceived barriers of health care access, which coded as “1”. On the other hand, if a woman didn’t report challenges of the obstacles mentioned above, like obtaining money, distance, companionship, and permission, it was considered no perceived barrier to health care access, coded as “0” [3, 12].

### Data management and analysis

The data were weighted using sampling weight, primary sampling unit, and strata before any statistical analysis to restore the representativeness of the survey and take into account the sampling design to get reliable statistical estimates. Descriptive and summary statistics were conducted using STATA version 14 software. To identify factors associated with the perceived barriers of health care access among reproductive-age women, generalized estimating equation (GEE) model was fitted [25]. The data nature of EDHS showed that women were nested within a cluster, and those who reside within the same clusters were correlated to each other compared to the other clusters. This data showed that the Intra Class Correlation (ICC) was calculated and found to be 40% indicated a correlation among observations in the clusters. These violated the conditional logistic regression model’s assumptions that consider the independence of observations and equal variance across clusters. The Generalized Estimating Equation (GEE) model was fitted with a logit link function and binomial family and working correlation structures of independent, exchangeable, unstructured, and autoregressive compared simultaneously. The model with the smallest standard error differences between robust and model-based standard errors that were the model with the exchangeable correlation structure was selected. To assess the strength of association between outcome and independent variables crude and adjusted odds ratio with a 95% confidence interval (CI) computed and presented on the table. Variables had less than 5% *p*-value in the multivariable GEE model considered independent factors associated with the perceived barriers of health care access.

### Model comparison

In this study, we fitted two models GEE which is a marginal model that considers correlation among clusters and a conventional logistic regression model with a robust standard error that also controlled for within-cluster correlations. To select the best-fitted model, we used quasi information criteria (QIC) for the model comparison and the result presented on the Table 1 as follow.

**Table 1:** Model comparison

Types of the model fitted	QIC
GEE	18,301
Conventional logistic	18,441

The GEE had the smallest QIC it better fits the data than the conventional logistic regression model.

## Result

### Socio-demographic characteristics

A total of 15,683 reproductive-age women were included in the final analysis of the study. The median age of women was 27 with (IQR: 20 to 35) years; nearly two-thirds (67.5%) of women gave birth previously, and 18 years is the median age to the first birth. More than three-four (77.8%) women were rural dwellers, 47.8% had no formal education, 63.8% were married, 46.3% rich wealth index, and 43.2% were orthodox Christians. The majority (94.7%) of women had no health insurance coverage; among interviewed women, 7.2% of them were pregnant during data collection, and 41.6% of them visited health facilities in the past 12 months (Table 2).

**Table 2:** Socio demographic and reproductive characteristics of women who aged 15–49 years in Ethiopia, 2016 (*n* = 15,683)

Characteristics	Category	Frequency	Percentage
Age in years	15–19	3381	21.6
	20–34	8064	51.4
	35–49	4238	27
Residence	Urban	3476	21.2
	Rural	12,207	77.8
Religion	Orthodox	6786	43.3
	Muslim	4893	31.2
	Protestant	3674	23.4
	Other	330	2.1
Education level	No formal education	7498	49
	Primary school	5490	35
	Secondary school	1818	11.5
	Diploma and above	877	5.5
Marital status	Never married	4037	25.7
	Married/living together	10,223	65.2
	Divorce/widowed/separated	1423	9.1
Wealth index	Poor	5442	34.7
	Middle	2978	19
	Rich	7263	46.3
Gave birth in the last five years	Yes	7590	48.4
	No	8093	51.6
Place of delivery (*n* = 7590)	Home	5066	66.7
	Health facility	2524	33.3
Had ANC follow up(*n* = 7590)	Yes	2818	37.1
	No	4772	63.9
Contraceptive use and intention	Yes	3974	25.34
	No	11,708	74.66
Visited health facility in the last 12 months	Yes	6526	41.6
	No	9157	58.4
Sex of household head	Male	11,960	76.3
	Female	3723	23.7
History of abortion	No	14,447	92.1
	Yes	1326	7.9
Working status	Working	5220	33.3
	Not working	10,463	66.7
Ever heard of fistula	Yes	5990	38.4
	No	9625	61.6
Health insurance coverage	Yes	830	5.3
	No	14,853	94.7
Currently pregnant	No	14,547	92.8
	Yes	1135	7.2

### Correlation between perceived barriers of health care access and reproductive health services

The chi-square analysis showed a correlation between reproductive health service (contraceptive utilization history, place of delivery, and previous ANC follow-up). The reproductive variables cannot be included in the GEE marginal analysis model because no use of these services may not be an indicator of health care perceived barriers (Table 3).

**Table 3:** The association between perceived barriers and reproductive health services using EDHS 2016

Reproductive health services		perceived barriers of health care access		Chi-square(1)	*p*-value
		Big problem	Not a big problem		
Contraceptive utilization history	Yes	8051	5151	252	< 0.001
	No	2033	448		
Place of delivery for the recent child	Institution	1351	1247	471	< 0.001
	Home	3493	902		
Had previous ANC follow up	Yes	3011	1701	252	< 0.001
	No	2033	448		

### Perceived barriers of health care access among reproductive-age women

In this study, more than two-thirds (69.9, 95%CI: 69.3 to 70.7) of reproductive-age women had at least one perceived barrier to access health care, of which money (54.8%), the distance of health facilities (50.3%) were the most frequently mentioned challenges. Furthermore, out of the currently pregnant women during the data collection time, about 73% of them had perceived barriers to healthcare access. Of the parameters used to assess perceived barriers of healthcare access, about 21.5% of women had multiple challenges (money, distance, companionship, and permission) (Fig. 1).

### Factors associated with perceived barriers of health care access

The result of the bi-variable analysis showed that all explanatory variables were associated with healthcare access’s perceived barriers at a 20% level of significance. The multivariable generalized estimated equation (GEE) model showed that variables like residence, marital status, age group, educational level, wealth index, and health insurance were significant determinants of perceived barriers of health care access at a 5% level of significance (Table 4).

**Table 4:** Bivariable and multivariable generalized estimating equation (GEE) regression analysis reproductive age group women in Ethiopia, 2016 (*n* = 15,683)

Characteristics	Health care access problem		Crude odds ratio (95%CI)	Adjusted OR (95%CI)
	Yes	No		
**Residence**				
Urban	1584	1892	1	1
Rural	9392	2814	4.00 (3.40, 4.70)	2.13 (1.79,2.53)*
**Household head**				
Male	8466	3495	1	1
Female	2511	1211	1.11 (1.04,1.19)	1.05 (0.97,1.11)
**Marital status**				
Married/living together	7345	2877	1	1
Never married	2592	1443	0.98 (0.91,1.06)	1.13 (0.95,1.25)
Divorced/widowed/ separated	1038	384	1.37 (1.22,1.54)	1.34 (1.17,1.54)*
**Age group**				
15–19	2291	1089	1	
20–34	5582	2481	1.014 (0.94,1.09)	1.06 (0.99,1.23)
35–49	3103	1135	1.21 (1.11,1.32)	1.24 (1.09,1.40)*
**Level of education**				
Diploma and above	349	527	1	1
No formal education	5847	1650	2.74 (2.37,3.17)	2.30 (1.95,2.72)*
Primary education	3906	1584	1.99 (1.74,2.28)	1.84 (1.58,2.15)*
Secondary education	873	943	1.33 (1.17,1.52)	1.31 (1.13,1.51)*
**Working status**				
Working	3407	1812	0.96 (0.90,1.02)	0.99 (0.92,1.06)
Not working	7569	2893	1	1
**Wealth status**				
Rich	4101	3161	1	1
Poor	4574	867	2.72 (2.46,3.02)	2.09 (1.86,2.35)*
Middle	2300	677	1.92 (1.71,2.16)	1.57 (1.38,1.79)*
**Health insurance**				
Insured	460	369	1	1
Non-insured	10,551	4337	1.27 (1.05,1.53)	1.19 (1.01,1.45)*
**Gave birth in the last five years**				
Yes	3288	2804	1	1
No	5688	1901	1.07 (1.01,1.14)	1.09 (0.98,1.19)
**Ever heard of fistula**				
Yes	7395	2229	1	1
No	3521	2468	0.73 (0.68,0.78)	0.79 (0.75,1.05)

* shows statistical significan;ce at *p*-value less than 0.05

Those women who reside in rural areas were 2.13 times more likely to had perceived barriers of health care access than urban residents (AOR = 2.13, 95%CI: 1.79 to 2.53). Similarly, women aged 35–49 years, the odds of perceived barriers to access health care were 1.24 times higher than those aged 15–19 years (AOR = 1.24, 95%CI: 1.09 to 1.40). The likelihood of having perceived barriers to access health care among divorced/separated women was increased by 34% compared to married/live together (AOR = 1.34, 95%CI: 1.17 to 1.54). Similarly, the odds of perceived challenges of health care access to those who had no formal education (AOR = 2.30, 95%CI: 1.95 to 2.72), primary (AOR = 1.84, 95%CI: 1.58 to 2.15) and secondary (AOR = 1.31, 95%CI: 1.13 to 1.51) higher compared to those who attended college and above. For women who had poor and middle wealth status, the odds of perceived barriers of health care access were 2.09 and 1.57 times higher, compared to wealthier women, respectively (AOR = 2.09, 95% CI: 1.86, 2.35) and (AOR = 1.57, 95% CI: 1.38, 1.79). Those women who had no health insurance coverage, the odds of perceived barriers of health care access increased by 19% compared to those insured (AOR = 1.19, 95% CI: 1.01, 1.45) (Table 3).

## Discussion

This study revealed that about 70% of women of reproductive age had perceived barriers of health care access due to at least one or more of the four reasons, of which difficulty of obtaining money and distance from health facilities were the most frequently mentioned barriers. The present study magnitude was significantly lower than previous EDHS reports of (95.7%) in 2005 [26] and (93.6%) in 2011 [27]. The Ethiopia government has made tremendous efforts to achieve millennium development goals of reducing maternal and child mortality, which might contribute to lower perceived barriers of health care among women. Additionally, the country’s economic growth in the last 15 years and it’s policy revision to provide basic maternal and child health services free of charge as exempted by the government may also contribute to the decline of perceived barriers among women [22, 23, 28].

However, the current study result showed a higher magnitude of perceived barriers, despite the global efforts for universal health access for all world peoples. Moreover, this finding was more elevated than study reports from Tanzania 65% [12] and 64.5% in South Africa [29]. This could be explained socio-cultural and economic difference among countries which may affect health-seeking behaviors. In general, despite 100% health facility coverage per population, different barriers are responsible for timely access and utilization of health care services among reproductive-age women, which made doubts about the achievement of SDGs universal reproductive health access and equitable distribution of services.

Personal and organizational characteristics attributed w with the perceived barriers of health care access among reproductive-aged women. Thus, women who reside in rural areas, the odds of perceived barriers of health care access were two times higher than in urban dwellings. This finding was consistent in previous studies in Tanzania, Ghana, and South Africa [12, 16, 29]. These could be due to the fact rural areas are associated with lower geographical accessibility of health facilities. Besides economic problems, there are also socio-cultural issues related to lower male involvement and support for women’s healthcare access. Older age (35–49 years) women were associated with increased perceived barriers of health care access compared to Youngers. This could be because women of the older generation may be affected by distance travel to obtain healthcare in peripheral areas. Also, financial hardship and dependency are higher at an older age compared to younger ones.

Divorced/separated women had increased barriers to health care access compared to a married one. This finding was in line with previous studies [12, 18, 19]. These could be explained by those married women who may have better economic and psychosocial support from their partners to access health care [30]. Indirectly, married women may have decided collectively to control their family size and fertility behavior, which could impact the health care access of a woman.

Similarly, women had no health insurance coverage associated with increased odds of health care access problems. This finding was consistent with previous studies [31]. Community-based health insurance has been implemented in Ethiopia since 2010 that protects the individual from unexpected catastrophic expenditure and minimize difficulties in obtaining money for consultation of the doctor [31]. Likewise, lower educational status below is associated with increased perceived barriers of health care access among women of reproductive age. Better educational levels may improve awareness and increase health-seeking behavior among reproductive-age women. This finding was consistent with other studies [12, 16, 19, 32].

This work also reported that Women who had financial hardships like poor and middle wealth class associated with an increased perceived barrier of healthcare access compared to riches. This finding was consistent with the previous study conducted in Tanzania [12]. These possible reasons might be that women who had a better wealth index may help them access health care for their best health outcomes. Besides, a better wealth index may reduce the difficulties of obtaining money to access health care.

This study has strengths of nationally representative data, and advanced statistical models were used to account correlations within clusters. However, this study has limitations of the survey’s cross-sectional nature, and spatial variability was not assessed. Also, the effects of the health system and health care worker factors were not addressed.

## Conclusions

A significant proportion of reproductive-age women faced barriers of health care access, of which, money and distance were the commonly perceived barriers. Divorced/separated marital status, old age, rural dwelling, no health insurance coverage, low economic situation, and level of education were factors associated with perceived barriers of health care access. These findings suggest that further strengthening and redistribution of health care services to those with low socio-economic status for the attainment of universal health coverage and equity of health.

## Data Availability

The data analyzed for this study is from the Ethiopian Demographic and Health Survey and accessible with permissions from the measure DHS.

## Abbreviations

ANC: Antenatal Care
AOR: Adjusted Odds Ratio
CI: Confidence Interval
EA: Enumeration Areas
EDHS: Ethiopia Demography and Health Survey
GEE: Generalized Estimating Eq.
ICC: Intra Class Correlation
IQR: Interquartile range
IR: Individual Record
MDG: Millennium Development Goals
QIC: Quasi-likelihood Information Criteria
SDG: Sustainable Development Goals
